# Paradoxical myopic shift following cycloplegia in retinopathy of prematurity patients: a case series

**DOI:** 10.4076/1757-1626-2-8970

**Published:** 2009-08-25

**Authors:** Nikolas JS London, Susan M Carden, William V Good

**Affiliations:** 1 Department of Ophthalmology, California Pacific Medical Center2340 Clay St. 5th Floor, San Francisco, CA 94115USA; 2 Smith Kettlewell Eye Research Institute2318 Fillmore St, San Francisco, CA 94115USA; 3 Department of Ophthalmology, Royal Children’s Hospital, Melbourne, Victoria, 3052Australia

## Abstract

**Introduction:**

Spectacle non-compliance is a significant problem in pediatric patients, and may have a variety of consequences. Non-compliance with myopic refractive correction could be secondary to a variety of issues, including age, discomfort, gender, urban vs. rural residence, presenting visual acuity, and degree of refractive error. We observed a phenomenon in our pediatric patients with retinopathy of prematurity that may add another possible explanation: incorrect prescription due to measures of increased, rather than decreased, myopia after cycloplegia.

**Case presentation:**

An unmasked, prospective study of 8 consecutive patients seen in a single practice. Retinoscopic refraction measurements were obtained before and after pharmacologic cycloplegia.

In all 13 eyes, there was either no change (2 eyes) or a myopic shift (11 eyes) in the measured refractive error. The average change in refraction was -1.58 and -1.54 for the right and left eyes, respectively (range 0 to -3.00 OD and 0 to -3.00 OS).

**Conclusions:**

The contribution of ocular components to refractive status differs between ROP and non-ROP eyes. Unanticipated myopic shift following cycloplegia in ROP patients may result in inappropriate glasses prescription with poor correction of visual acuity. This may contribute to spectacle noncompliance in this group.

## Introduction

Spectacle non-compliance is occasionally encountered in pediatric patients [1,2], even though refractive errors may have significant functional, economic, medical, and educational consequences. Non-compliance with myopic refractive correction could be secondary to a variety of issues, including age, discomfort, gender, urban vs. rural residence, presenting visual acuity, and degree of refractive error [2,3]. In pediatric patients with retinopathy of prematurity (ROP), we found increased rather than decreased myopia after cycloplegia. This finding may be of interest to ophthalmologists who care for children with resolved, advanced ROP.

## Case presentation

### Methods

Ethics committee approval was obtained from the Smith Kettlewell Eye Research Institute. We collected our data in a prospective, but unmasked fashion. The cases were accumulated from 8 consecutive ROP patients seen by one author (WVG) in his office, with no others left out (Table 1). Informed consent for participation was obtained for each patient from the patients' parents. None of these children participated in the Early Treatment for Retinopathy of Prematurity Study. The patients ranged in age from 8 months to 7 years, with an average age of 41 months. For each patient, manifest and cycloplegic refractions were measured in both eyes, and the difference between the two measurements was determined. Cycloplegia was accomplished with tropicamide 1%. Measurements were obtained using a retinoscope (Welch Allyn, Skaneateles Falls, NY, USA) from 13 of 16 eyes, limited in 3 eyes by stage 5 disease.

**Table 1.** Summary of data for all patients indicating age and manifest (MRx) and cycloplegic (CRx) refractive error measurements in right and left eyes

Patient #	Age	MRx OD	MRx OS	CRx OD	CRx OS
1	4 years	-5.25	-4.00	-6.25	-4.50
2	8 months	1.50	1.25	-1.50	-1.75
3	6 years	-8.75	stage 5	-9.50	stage 5
4	16 months	-1.75	-1.75	-4.00	-4.00
5	7 years	stage 5	-7.50	stage 5	-7.50
6	3 years	stage 5	-19.00	stage 5	-22.00
7	3 years	-7.50	-4.50	-8.00	-5.00
8	2 years	-8.50	-7.50	-10.50	-9.00

MRx, manifest refraction; CRx, cycloplegic refraction; OD, right eye; OD, left eye.

### Results

In all 13 eyes, there was either no change (2 eyes) or a myopic shift (11 eyes) in the measured refractive error. The average change in refraction was -1.58 and -1.54 for the right and left eyes, respectively (range 0 to -3.00 OD and 0 to -3.00 OS).

## Discussion

Cycloplegic refraction should provide the most hyperopic refraction possible for an individual, because cycloplegic agents paralyze muscles involved in accommodation. Studies on the effect of cycloplegia have not evaluated children who recovered from advanced ROP, however.

The shift toward myopia after cycloplegia in children who had advanced ROP can be explained by a difference in the contribution of ocular components to refractive status differs between ROP and non-ROP eyes. In patients without ROP, the refractive state of the eye is most strongly correlated with the axial length of the eye, less so with corneal curvature or anterior chamber depth, and not correlated with the power of the lens [4,5]. In children with a history of advanced ROP, axial length does not change much, and myopia is primarily lenticular in etiology [6,7]. The lens edge is more refractive than its center, and the lens may be more spherical and anteriorized, due to peripheral ROP retinal cicatricial changes. Lenticular myopia is noted even in normal adult eyes in dim lighting and after cycloplegia [8-10].

Following recovery from ROP, it is likely that the affected eye has a new constellation of factors that contribute to refractive status. This study looks at cases of myopia after cycloplegia, and is not controlled. Nevertheless, attention should be given to the manifest refraction in infants who had advanced ROP. In some instances, paradoxical myopia could explain spectacle non-compliance. This study has several limitations. Scientific rigor is limited in uncontrolled case series. Also we have a relatively small cohort of eight patients. Moreover, we have no evidence to support our assertion that an incorrect spectacle prescription is associated with non-compliance. However, this phenomenon has not been reported previously, and should be considered when measuring glasses ROP patients.
